# Single-cell Transcriptomics in Autoimmune Diseases: Mechanistic Insights, Cross-disease Convergence, and Translational Challenges in Rheumatoid Arthritis, Systemic Lupus Erythematosus, Type 1 Diabetes, and Multiple Sclerosis

**DOI:** 10.1007/s12016-026-09211-0

**Published:** 2026-10-14

**Authors:** Thanh-Hong Nguyen, Tuan-Dinh Nguyen, Oanh-Thi Hoang, Thuy-Thu T. Can, Minh-Huyen T. Nguyen, Mai-Hoang Tran, Hieu-Chi Chu, Dinh-Van Nguyen

**Affiliations:** 1 Vinmec-VinUni Institute of Immunology - College of Health Sciences - VinUniversity - Vinhomes Ocean Park, Gia Lam, Hanoi Vietnam; 2 Innovations in Health Sciences, VinUniversity, Hanoi, Vietnam; 3 Department of Medical Genetics and Rare Diseases, Vinmec Times City International Hospital - Vinmec Healthcare System, 458 Minh Khai, Hai Ba Trung, Vinh Tuy, Hanoi Vietnam; 4 Centre of Allergy and Clinical Immunology, Bach Mai Hospital, Hanoi, Vietnam; 5 Centre of Allergy and Clinical Immunology - Vinmec Times, City International Hospital - Vinmec Healthcare System, 458 Minh Khai, Hai Ba Trung, Vinh Tuy, Hanoi Vietnam

**Keywords:** Single-cell RNA sequencing, Autoimmune diseases, Rheumatoid arthritis, Systemic lupus erythematosus, Lupus nephritis, Type 1 diabetes, Multiple sclerosis, Precision medicine

## Abstract

Autoimmune diseases are characterized by loss of immune tolerance and substantial heterogeneity across patients, tissues, and disease stages. Although bulk transcriptomic studies have identified important inflammatory pathways, they cannot directly resolve rare, transitional, and tissue-contextualized cell states that may be relevant to disease mechanisms and therapeutic response. Single-cell RNA sequencing (scRNA-seq) enables transcriptomic profiling at cellular resolution, while complementary multimodal, receptor-repertoire, epigenomic, and spatial approaches can link cellular states to protein phenotype, clonal architecture, regulatory programs, and tissue context. This review critically examines recent single-cell studies in rheumatoid arthritis, systemic lupus erythematosus and lupus nephritis, type 1 diabetes, and multiple sclerosis. Rather than providing a descriptive catalogue of published studies, we assess the strength of available evidence by distinguishing independently replicated findings from observations supported by limited studies, and cross-sectional associations from longitudinal, treatment-response, and functional evidence. Across the selected diseases, studies repeatedly report inflammatory programs involving interferon signaling, chemokine-mediated immune recruitment, and stromal-immune interactions; however, evidence for convergence at the cell-state and regulatory-network levels remains limited, and the extent to which shared signatures represent common pathogenic mechanisms rather than inflammatory context remains uncertain. We evaluate the translational potential of scRNA-seq for biomarker discovery, patient stratification, and treatment response prediction, while considering evidence from related tissue-based molecular stratification approaches, including the R4RA randomized trial in rheumatoid arthritis. R4RA provides prospective randomized evidence that synovial molecular features can enrich for differential therapeutic responses, but its use of bulk synovial transcriptomics and histopathology does not constitute prospective validation of single-cell-derived biomarkers. We further highlight cohort biases, reproducibility challenges, dissociation artifacts, computational ambiguity, and privacy concerns that constrain immediate clinical implementation. Together, these advances position single-cell profiling as a mechanistic discovery platform with substantial translational promise, contingent on standardization, validation across diverse cohorts, and integration with functional perturbation approaches.

## Highlights

Single-cell RNA sequencing resolves pathogenic immune and stromal cell states that are masked in bulk transcriptomic analyses.

In rheumatoid arthritis, systemic lupus erythematosus and lupus nephritis, type 1 diabetes, and multiple sclerosis, single-cell studies have refined our understanding of disease-associated biological processes and identified candidate disease-associated cell states, though most findings require independent replication and functional validation.

Multimodal approaches (CITE-seq, SCITO-seq, scTCR/scBCR-seq, scATAC-seq, spatial transcriptomics) increase biological interpretability but introduce new analytical, standardization, and ethical challenges.

Cross-disease comparisons identify shared autoimmune programs-particularly interferon signaling, chemokine networks, and tissue-immune crosstalk - but the level of convergence (pathway, cell-state, or regulatory network) differs across diseases and is often overstated.

Prospective randomized evidence supports tissue-based molecular stratification in RA (R4RA), whereas clinical validation of single-cell-derived biomarkers remains preliminary; broader implementation will require standardization, longitudinal validation, equitable cohort representation, and integration with functional perturbation approaches.

## Introduction

Autoimmune diseases are chronic inflammatory disorders in which immune tolerance to self-antigens is lost, leading to sustained immune activation and tissue injury [1, 2]. They share recurring features-immune-cell dysregulation, cytokine imbalance, aberrant antigen presentation, and failure of resolution pathways - but treatment selection is still guided more by clinical phenotype than by mechanism [2, 3]. This gap reflects biological heterogeneity: similar clinical syndromes may arise from distinct cellular programs that bulk transcriptomics cannot resolve [4].

Single-cell RNA sequencing (scRNA-seq) addresses this limitation by profiling gene expression in individual cells, enabling identification of rare populations, reconstruction of differentiation trajectories, and mapping of inflamed-tissue ecosystems [5, 6]. In autoimmune research, scRNA-seq has identified pathogenic fibroblast states in rheumatoid arthritis (RA) [7], interferon-driven immune and renal cell programs in lupus nephritis (LN) [8], stem-like autoreactive T-cell populations in type 1 diabetes (T1D) [9], and compartmentalized inflammatory niches in multiple sclerosis (MS) [10]. Multimodal and complementary single-cell extensions, including CITE-seq, SCITO-seq, scTCR-seq, scBCR-seq, scATAC-seq, and spatial transcriptomics, link transcriptional programs to protein phenotype, clonal expansion, regulatory architecture, and tissue context, moving the field toward mechanistic classification [11–15].

Several recent reviews have summarized the expanding applications of single-cell technologies in autoimmune disease research and provided a valuable overview of disease-specific findings [16–18]. Building on these contributions, the present review emphasizes three distinctions that are particularly important for interpreting the field: (1) reported disease-associated cell states vary in their degree of replication across cohorts and platforms; (2) cross-disease convergence may occur at the level of pathways, cellular states, or regulatory networks, which carry different biological and therapeutic implications; and (3) most proposed single-cell-derived biomarkers remain at an exploratory or proof-of-concept stage rather than being ready for routine clinical implementation. Accordingly, this review focuses on the robustness of current single-cell discoveries, the level of evidence supporting cross-disease convergence, and the extent to which single-cell profiling is moving autoimmune disease research toward precision medicine.

## Review Methodology

This review was conducted as a critical narrative review aimed at synthesizing current evidence on the application of single-cell technologies in autoimmune diseases while critically evaluating the strength, reproducibility, and translational relevance of published findings. The review focused on four representative autoimmune diseases - rheumatoid arthritis (RA), systemic lupus erythematosus/lupus nephritis (SLE/LN), type 1 diabetes (T1D), and multiple sclerosis (MS) - because they represent distinct target organs, immune mechanisms, and levels of single-cell evidence.

### Literature Search

A structured and iterative literature search was performed using PubMed/MEDLINE, Web of Science, and Google Scholar. Searches were conducted through June 30, 2026, with the primary search window covering studies published from December 1996 onward, together with seminal earlier studies where necessary to provide historical context. Search terms included combinations of “single-cell RNA sequencing”, “scRNA-seq”, “single-cell transcriptomics”, “single-cell multi-omics”, “CITE-seq”, “SCITO-seq”, “scATAC-seq”, “spatial transcriptomics”, “single-cell TCR sequencing”, “single-cell BCR sequencing”, “rheumatoid arthritis”, “systemic lupus erythematosus”, “lupus nephritis”, “type 1 diabetes”, “multiple sclerosis”, “autoimmune disease”, “immune atlas”, and “precision medicine”. Reference lists of relevant reviews and original articles were also screened to identify additional studies.

### Study Selection and Evidence Appraisal

This review was designed as a critical narrative review rather than a systematic review or meta-analysis, study selection involved structured database searching supplemented by reference-list screening and targeted identification of landmark studies and emerging evidence. Studies were selected using predefined eligibility considerations consistent with the objectives of this critical narrative review. **Inclusion criteria** comprised: (1) peer-reviewed primary research articles reporting original single-cell or spatially resolved molecular data; (2) studies involving rheumatoid arthritis (RA), systemic lupus erythematosus/lupus nephritis (SLE/LN), type 1 diabetes (T1D), or multiple sclerosis (MS), with studies of other immune-mediated diseases considered selectively when they informed broader cross-disease themes; (3) studies using single-cell RNA sequencing (scRNA-seq), single-cell multi-omics, CITE-seq, scATAC-seq, single-cell TCR/BCR sequencing, spatial transcriptomics, or closely related single-cell technologies; and (4) studies addressing disease mechanisms, cellular heterogeneity, molecular signatures, disease stratification, treatment response, biomarkers, or other aspects relevant to clinical translation. Particular emphasis was placed on studies involving human tissues, longitudinal or prospective clinical cohorts, and datasets with appropriate clinical or phenotypic annotation.

**Exclusion criteria** included studies without relevant single-cell or spatial molecular data; studies focused exclusively on methodological development without substantive application to autoimmune disease; non-peer-reviewed publications and conference abstracts lacking sufficient primary data; and publications that did not provide information relevant to the biological, clinical, or translational questions addressed in this review. Review articles were not used as primary evidence for mechanistic or translational conclusions when relevant original studies were available, but were consulted for background, historical context, reference tracking, and identification of emerging areas of research.

Selection was not based solely on publication number or citation frequency. Priority was given to studies that provided detailed descriptions of patient characteristics and sample collection, clearly defined comparison groups, adequately described single-cell or spatial experimental and analytical procedures, appropriate biological replication, and independent or orthogonal validation when available. Studies were also prioritized when their findings directly addressed disease biology, patient heterogeneity, biomarkers, therapeutic targets, or clinically relevant stratification. Landmark consortium efforts, including the Accelerating Medicines Partnership (AMP), the Human Cell Atlas (HCA), and the Human BioMolecular Atlas Program (HuBMAP), were also considered when they provided reference atlases, large-scale datasets, or methodological resources directly relevant to the questions addressed in this review. These criteria were applied qualitatively by the authors to determine the relevance and evidentiary contribution of individual studies; no formal numerical study-quality or risk-of-bias score was assigned.

**Fig. 1:**
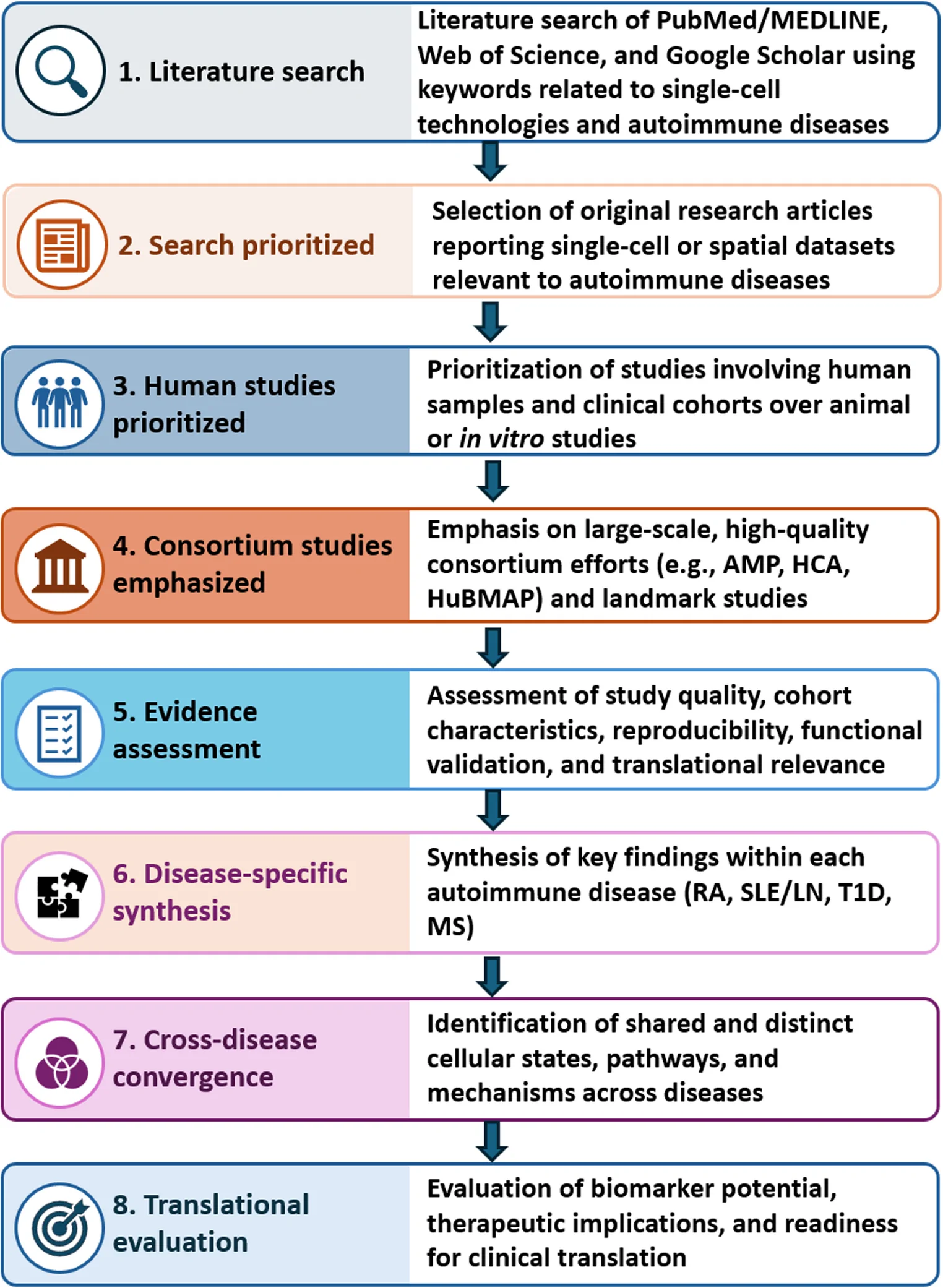
Systematic framework for synthesizing single-cell and spatial transcriptomic evidence in autoimmune diseases

Figure 1 illustrates the structured workflow used to identify, screen, and synthesize relevant single-cell studies across the four diseases, with particular attention to study design, tissue source, analytical modality, replication, functional validation, and translational evidence.

The eight-step workflow begins with a literature search of PubMed/MEDLINE, Web of Science, and Google Scholar using keywords related to single-cell technologies and autoimmune diseases. Original research articles reporting single-cell or spatial datasets relevant to autoimmune diseases are then prioritized, with emphasis on studies involving human samples and clinical cohorts over animal or in vitro studies. Large-scale, high-quality consortium datasets - including AMP, HCA, and HuBMAPas well as landmark studies are specifically emphasized. The included studies are assessed for methodological quality, cohort characteristics, reproducibility, functional validation, and translational relevance. Key findings are subsequently synthesized for individual autoimmune diseases, including rheumatoid arthritis (RA), systemic lupus erythematosus and lupus nephritis (SLE/LN), type 1 diabetes (T1D), and multiple sclerosis (MS). Cross-disease convergence is then evaluated to identify shared and disease-specific cellular states, pathways, and mechanisms. Finally, the translational potential of the findings is assessed, including biomarker potential, therapeutic implications, and readiness for clinical translation. AMP, Accelerating Medicines Partnership; HCA, Human Cell Atlas; HuBMAP, Human BioMolecular Atlas Program.

### Rationale for Disease Selection and Characterization of the Evidence Base

**The four diseases selected** - rheumatoid arthritis (RA), systemic lupus erythematosus/lupus nephritis (SLE/LN), type 1 diabetes (T1D), and multiple sclerosis (MS) - represent complementary autoimmune contexts with distinct target organs, immune mechanisms, tissue environments, and levels of single-cell evidence and translational maturity. This focused selection enables critical comparison of evidence strength, reproducibility, and translational relevance across diseases. Other autoimmune and immune-mediated diseases, including Sjögren’s disease, inflammatory bowel disease, psoriasis, and ankylosing spondylitis, are not comprehensively covered; their omission reflects the need for depth and cross-disease synthesis rather than an exhaustive catalogue. Findings from these and other diseases are considered relevant to broader shared cellular and molecular programs

#### Representative primary-study evidence base

To characterize the evidence base underlying the cross-disease comparison, we identified the representative primary studies cited and discussed for each selected disease and distinguished these from review articles, methodological papers, editorials, and other contextual references. The representative primary studies were further characterized according to the principal tissue or biospecimen analyzed and the single-cell or spatial profiling approaches employed. Across the four diseases, the evidence encompasses synovial tissue and synovial biopsies in RA; peripheral blood and renal tissue/biopsies in SLE/LN; pancreatic islets and pancreatic/lymphatic tissues in T1D; and cerebrospinal fluid, peripheral blood, and post-mortem central nervous system tissues in MS. The primary studies specific to each disease group are summarized in Table 1, including the number of studies, tissue sources, sequencing modalities, and representative references

**Table 1:** Overview of Primary Bulk transcriptomics, Single-Cell and Spatial Transcriptomic Studies Across Autoimmune Diseases

Disease object	Total number of primary studies	Tissue sources	Sequencing modalities	Reference
Rheumatoid arthritis (RA)	**10**	Primarily synovial tissue/synovium; some studies include isolated synovial fibroblasts or macrophages	scRNA-seq; scRNA-seq integrated with mass cytometry; three-dimensional spatial transcriptomics	[7, 19–27]
Systemic Lupus Erythematosus (SLE) / Lupus Nephritis (LN)	**7**	Peripheral blood/PBMCs; Kidney biopsy tissue	Bulk/modular transcriptomics; scRNA-seq; Single-cell multi-omic/population-scale atlas	[8, 28–33]
Type 1 Diabetes Mellitus (T1D)	**9**	Human pancreatic islets; pancreas-draining lymphatic tissue; pancreatic islets/immune cells	Single-cell multi-omics; scRNA-seq; single-nucleus RNA-seq	[34–42]
Multiple Sclerosis (MS)	**8**	Cerebrospinal fluid (CSF); brain/CNS lesion tissue; peripheral blood; peripheral blood + CSF	scRNA-seq; single-nucleus RNA-seq; integrated single-cell blood/CSF profiling; molecular/transcriptomic modeling	[43–50]

## Why Single-cell Analysis Matters in Autoimmune Disease

**Fig. 2:**
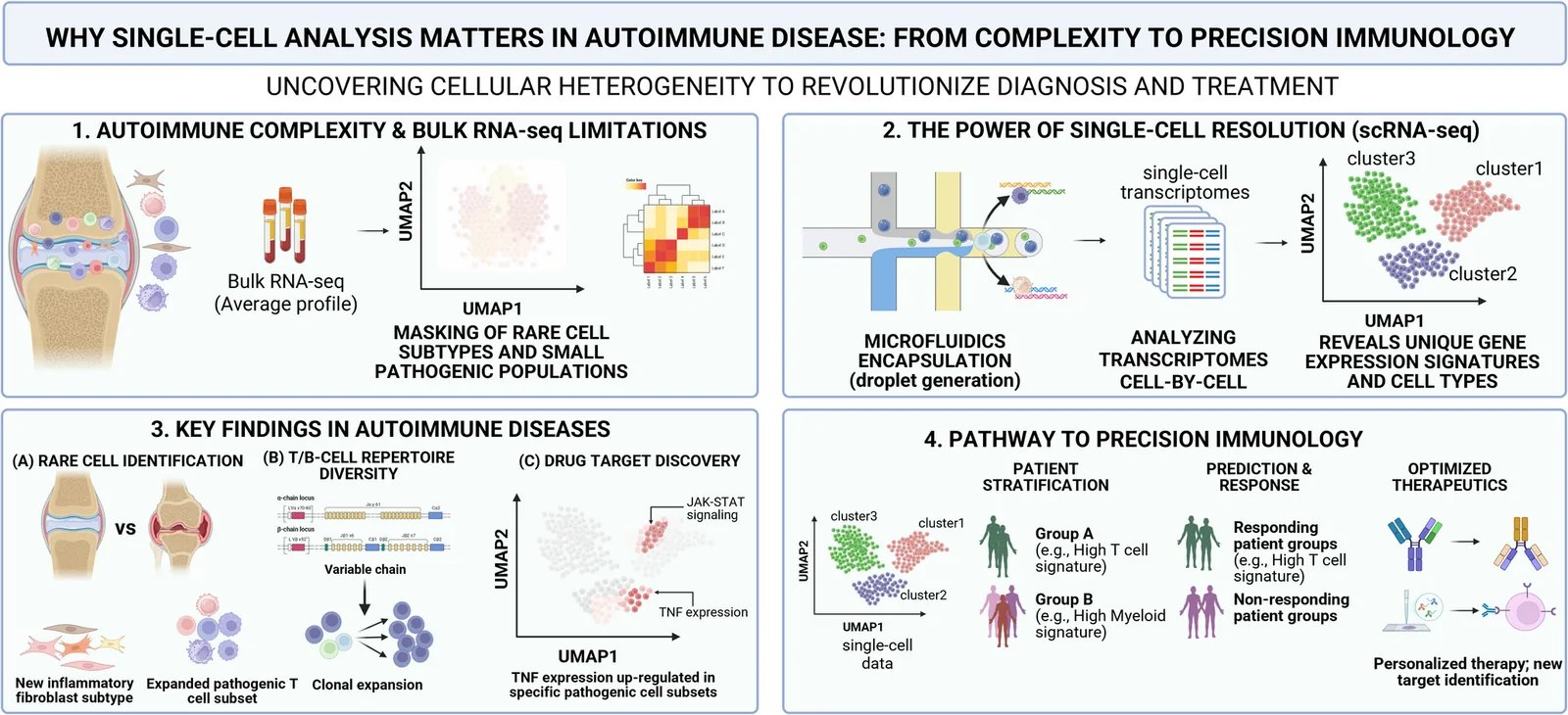
Why single-cell analysis matters in autoimmune disease: from complexity to precision immunology (Created by BioRender)

Autoimmune tissues comprise complex mixtures of lymphocytes, myeloid cells, stromal cells, endothelial cells, and parenchymal cells, many in disease-specific activation states. Pathogenic contribution depends not only on lineage but also on transient transcriptional state, clonality, differentiation stage, and spatial localization-features poorly captured by bulk assays. The analytical power of scRNA-seq, however, depends critically on platform choice, sample handling, and analytical framework, and accurate appraisal of disease findings requires explicit consideration of these dependencies. As schematized in Fig. 2, single-cell transcriptomics - especially when paired with surface epitope profiling, antigen receptor sequencing, chromatin accessibility, and spatial context - resolves rare disease-associated cell states, clonal dynamics, and tissue-stromal interactions that drive disease progression and define precision immunology targets.

Single-cell RNA sequencing (scRNA-seq) provides a high-resolution framework for dissecting the cellular heterogeneity underlying autoimmune diseases and enables the transition from conventional disease classification to precision medicine. (1) Autoimmune complexity and limitations of bulk RNA sequencing: autoimmune tissues contain diverse immune and stromal cell populations, whereas bulk RNA-seq measures only average transcriptomic signals, obscuring rare pathogenic subsets and cellular heterogeneity. (2) Power of single-cell resolution: droplet-based scRNA-seq profiles transcriptomes at the individual-cell level, allowing data-driven clustering and identification of distinct cell populations based on unique gene-expression signatures. (3) Key biological insights in autoimmune diseases: single-cell approaches have revealed previously unrecognized inflammatory fibroblast populations, expanded pathogenic T-cell subsets, clonal lymphocyte expansion through paired B-cell and T-cell receptor sequencing, and dysregulated signaling pathways and transcriptional programs enriched in specific disease-associated cell states [22, 51, 52]. (4) Translation to precision medicine: integration of single-cell data enables patient stratification into molecular endotypes, improves prediction of therapeutic responses, and supports the development of personalized treatment strategies and novel targeted therapies. Collectively, single-cell technologies are transforming the understanding, diagnosis, and treatment of autoimmune diseases by linking cellular heterogeneity to mechanism-based precision medicine.

### Platform Choices and their Impact on Biological Resolution

Single-cell workflows include tissue dissociation, cell or nucleus isolation, barcode-based library preparation, sequencing, and computational analysis [53–55]. Droplet-based platforms such as 10x Genomics Chromium are widely used for tissue atlasing because they profile tens of thousands of cells per experiment [53, 56, 57]. However, standard 3′ and 5′ gene expression chemistries capture only one end of each transcript and therefore provide limited information on full-length transcript structure and isoform diversity. Plate-based methods (Smart-seq2, Smart-seq3, as well as long-read sequencing approaches) offer greater per-cell sensitivity and improved characterization of transcript structure, full-length coverage but are limited to hundreds of cells [5, 58, 59]. Direct comparisons have shown that Smart-seq2 can detects more low-abundance and alternatively spliced transcripts, whereas droplet-based approaches provide substantially greater cellular throughput but with the high chance of increased dropout for low-expression genes [60].

Importantly, the ability to detect rare populations is not determined by platform choice alone. Higher-throughput droplet-based methods increase the probability of sampling low-frequency populations, but detection depends on the total number of cells captured, the frequency of the population, cell-type-specific recovery and survival, sample quality, sequencing depth, and downstream analytical criteria [61, 62]. For a population occurring at frequency *p* among *N* captured cells, approximately *N × p* cells would be expected before accounting for cell loss and quality-control filtering. Thus, a population representing 1% of captured cells would yield approximately 100 cells among 10,000 profiled cells, whereas a 0.1% population would yield only approximately 10 cells. Increasing cell numbers therefore improves sampling of rare populations but cannot compensate for systematic loss of fragile or poorly recovered cell types during tissue dissociation or processing [63]. Moreover, “rare” is context-dependent and should not be interpreted solely according to a fixed frequency threshold.

Critically, many major autoimmune tissue atlases - including AMP RA Phase 2 [21, 29], foundational AMP LN studies [29, 64], and chronic active MS lesion atlases [45, 47] - have relied substantially on 10x Chromium-based workflows. This methodological convergence facilitates cross-study integration and standardized analysis but also introduces shared technical constraints, including incomplete characterization of transcript isoforms and splice variants. Importantly, the number of cells profiled should not be equated with the number of independent biological observations: cells are nested within patients, and robust patient-level inference depends on adequate numbers of individuals as well as sufficient cells per patient. Unequal cell recovery across patients, differences in tissue dissociation, cryopreservation, processing time, and other pre-analytical factors can further influence cellular composition and transcriptional states and may contribute to technical or biological variation between datasets [63, 65, 66].

### Multimodal Extensions Enhance Biological Resolution but Require Careful Interpretation

CITE-seq couples transcriptomic profiling with oligonucleotide-tagged antibodies, enabling simultaneous quantification of mRNA and surface proteins and thereby improving the resolution of immune-cell phenotypes beyond transcriptomic information alone [14, 67, 68]. The added value of protein measurements, however, is not uniform across cell types. Protein-level information is particularly informative for immune populations, for which many lineage, differentiation, activation, and exhaustion states are defined by cell-surface molecules whose protein abundance may not be reliably inferred from transcript levels because of transcriptional - translational discordance, low transcript abundance, or post-transcriptional regulation [69]. Multimodal measurements can therefore improve discrimination among closely related immune populations and provide orthogonal evidence for cell-state annotation [69, 70]. In contrast, for some stromal populations, the incremental benefit of a limited surface-protein panel may be more context-dependent, as their identity and functional states are often more strongly captured by broad transcriptional programs involving extracellular-matrix organization, structural components, growth factors, and tissue-specific pathways [21]. Protein profiling can nevertheless remain valuable for selected stromal markers and for resolving populations with overlapping transcriptional states [21]. Thus, the utility of multimodal profiling should be considered cell-type- and question-dependent rather than assuming that protein measurements provide equivalent gains across all populations.

More recently, high-throughput derivatives such as SCITO-seq (single-cell combinatorial indexed cytometry sequencing) have been developed to increase scalability and reduce costs through combinatorial indexing strategies, potentially facilitating population-scale studies and large multicenter atlases [71]. These approaches are particularly attractive for autoimmune disease research, where longitudinal monitoring and the analysis of rare cellular populations often require profiling very large numbers of cells. Nevertheless, multimodal protein measurements remain inherently dependent on antibody panel design. Differences in antibody availability, validation, and panel composition limit comparability across studies, while the restriction to predefined targets may bias analyses toward established immunophenotypic frameworks and potentially reduce the discovery of previously unrecognized cellular states.

Single-cell T-cell receptor (scTCR-seq) and B-cell receptor (scBCR-seq) sequencing complement transcriptomic analyses by linking cellular states with clonal architecture and antigen receptor repertoires. Integration of receptor sequences with gene-expression profiles has provided important insights into clonal expansion, differentiation trajectories, and lymphocyte dynamics in autoimmune diseases [72–74]. However, repertoire characterization by droplet-based platforms is influenced by sampling depth and cell recovery, often favoring expanded clonotypes. Moreover, inference of antigen specificity from receptor sequences remains imperfect, as sequence similarity alone does not necessarily predict antigen recognition. Consequently, computational predictions generally require orthogonal functional validation before pathogenic significance can be established. Emerging approaches that combine receptor sequencing with antigen barcoding or peptide-MHC multimer technologies may help bridge this gap, although their application to autoimmune diseases remains relatively limited [75, 76].

### Epigenetic and Spatial Extensions Deepen Biological Context

scATAC-seq identifies regions of accessible chromatin and, when integrated with scRNA-seq data, enables the inference of regulatory networks and candidate transcription factors associated with disease states [12, 40]. However, these inferences remain correlative and require functional validation to establish causal relevance. Spatial transcriptomics (ST) preserves tissue architecture but spans platforms with very different resolutions [11, 77]. For instance, 10x Visium offers multi-cellular spot-level resolution (~ 55 μm), whereas single-molecule imaging approaches like MERFISH and seqFISH provide true single-cell resolution at the expense of genomic throughput. Because most autoimmune ST studies have relied on Visium, inferred cell-cell interactions require validation by higher-resolution modalities to confidently establish direct physical proximity.

Single-nucleus RNA sequencing (snRNA-seq) reduces dissociation-induced bias and facilitates transcriptomic profiling of difficult specimens, including brain tissue and archived FFPE samples [10, 36, 77]. It has been particularly valuable for autopsy-based multiple sclerosis atlasing [45, 47]. Nevertheless, snRNA-seq is not a universal replacement for whole-cell methods, as nuclear RNA profiling provides limited representation of cytoplasmic transcripts and certain immune receptor sequences and may incompletely capture dynamic post-transcriptional states.

### Reference Atlases Enable Comparative Cellular Annotation

Reference atlases, including the Human Cell Atlas, provide baseline maps of healthy tissues and immune cell states [78], facilitating the distinction between homeostatic cell populations and disease-associated activation states. Automated annotation frameworks, such as CellTypist, Azimuth, and scArches, leverage these references to support standardized cell-type assignment and have been incorporated into large-scale consortium workflows [69, 79]. Nevertheless, several limitations deserve consideration. First, autoimmune tissues frequently harbor disease-associated cell states - such as pathogenic fibroblast subsets in rheumatoid arthritis (RA), expanded extrafollicular B cells in systemic lupus erythematosus (SLE), and lipid-associated microglia in chronic active multiple sclerosis (MS) lesions - that are rare or absent in healthy reference datasets. Consequently, automated annotation methods may assign these populations to the closest reference-defined cell type, potentially obscuring disease-specific biology. Second, discrepancies among studies may arise not only from biological variation but also from differences in annotation strategies, clustering resolution, and reference atlas versions. Such methodological dependencies are rarely quantified and can complicate the assessment of reproducibility across datasets. The development of harmonized, disease-specific reference atlases, together with standardized annotation frameworks, will therefore be important for improving the consistency and interpretability of single-cell studies [69, 80].

### From Cellular States to Molecular Endotyping and Therapeutic Strategies

Beyond its technical capabilities, single-cell analysis has fundamentally altered the conceptual framework through which autoimmune diseases are understood. Traditional models, largely derived from bulk assays and candidate-based immunology, viewed disease pathogenesis in terms of dysregulation of broad cell lineages or individual cytokine pathways [81, 82]. In contrast, single-cell approaches reveal that autoimmune disorders are composed of dynamic and heterogeneous cellular ecosystems, in which discrete cell states, rather than canonical cell types alone, drive tissue inflammation and disease progression [31, 83]. Studies across rheumatoid arthritis, systemic lupus erythematosus, type 1 diabetes, and multiple sclerosis have shown that pathogenic transcriptional programs may be shared across multiple lineages and anatomical compartments, whereas cells with similar phenotypic labels can occupy distinct functional states [21, 84]. Single-cell analyses have also highlighted the importance of cellular interactions, spatial organization, and developmental trajectories, shifting attention from static cell classifications to dynamic networks of immune and stromal cells [85, 86]. Consequently, autoimmune diseases are increasingly viewed not as homogeneous clinical entities but as collections of evolving cellular states and regulatory circuits, providing a foundation for molecular endotyping and more mechanism-based therapeutic strategies [87, 88].

## Major Disease-specific Insights

### Rheumatoid Arthritis

Rheumatoid arthritis (RA) has emerged as one of the most extensively profiled autoimmune diseases at single-cell resolution. These studies have transformed the classical view of RA pathogenesis - from one centered on leukocyte infiltration and synovial hyperplasia to one of a highly organized cellular ecosystem, in which diverse immune and stromal cell populations, their transcriptional states, and their interactions vary across patients and contribute to disease heterogeneity [19–21]. Many influential findings, however, derive from a small number of consortiums study whose generalizability requires careful evaluation. Interpretation is further complicated by differences in age, sex, treatment exposure, and disease activity, each of which can influence immune and stromal cell states independently of disease classification. These variables should therefore be considered when comparing cellular phenotypes across cohorts, particularly when treatment exposure or disease activity is unevenly distributed between groups [22, 89].

The AMP RA/SLE program is the most influential synovial atlasing effort. AMP Phase 2 profiled > 314,000 cells from synovial tissue of 79 donors and stratified samples into six cell-type abundance phenotypes (CTAPs) including (1) endothelial, fibroblast and myeloid cells; (2) fibroblasts; (3) T cells and fibroblasts; (4) T and B cells; (5) T and myeloid cells; and (6) myeloid cells. This information provides one of the most comprehensive frameworks for molecular stratification of RA synovium [21]. However, several limitations should be noted. AMP cohorts predominantly comprise patients with active disease and prior exposure to disease-modifying or biologic therapies, which may limit generalizability of findings to patients with early-stage, treatment-naïve, or seronegative disease. Cohort representation is also skewed toward North American and European populations, while single-cell sample sizes (tens of patients). Although these sample sizes are well suited for discovering cellular states and tissue architectures, they remain limiting for robust patient stratification at a clinically validated level [21, 23].

A central finding is the reclassification of synovial fibroblasts from passive structural cells to active participants in disease pathogenesis [19, 21, 24]. Lining-layer fibroblasts (CD55 + or PRG4+) associate with matrix remodeling and protease expression; sublining fibroblasts (CD90/THY1+) link more strongly to leukocyte recruitment and inflammatory persistence [16, 19]. Notch3-expressing sublining fibroblasts have been proposed as candidate regulators of chronic synovitis, with murine work showing that endothelial Notch ligand-driven Notch3 signaling regulates perivascular sublining fibroblast differentiation [90]. Two important qualifications should be noted. First, synovial fibroblast nomenclature varies substantially across studies - Stephenson et al. initially described three subsets, Mizoguchi et al. defined distinct functional populations, and the AMP Phase 2 consortium subsequently identified a more granular subpopulation annotation [19, 21, 91].

Myeloid heterogeneity has been similarly refined. Rather than a uniform inflammatory infiltrate, the RA synovium contains multiple macrophages and monocyte-derived states with differing inflammatory, interferon-responsive, phagocytic, and reparative features [7, 16]. Alivernini et al. profiled synovial macrophages across early, treatment-refractory, and remission RA and identified MerTK+TREM2-high and MerTK+LYVE1 + subpopulations enriched in remission, with transcriptional programs favoring resolution of inflammation [7]. These observations suggest a shift in therapeutic thinking from suppression of inflammatory macrophages toward promotion of pro-resolving programs. These inferences are based primarily on cross-sectional comparisons across patients rather than longitudinal sampling of the same individuals. Consequently, current data cannot determine whether macrophage populations undergo dynamic state transitions within individual patients or whether distinct patients preferentially occupy different, relatively stable macrophage programs. Across studies, reproducibility is generally stronger at the level of transcriptional programs than at the level of individual cluster definitions.

Spatial transcriptomic studies further indicate that RA synovial inflammation is anatomically organized, with immune and stromal programs distributed across distinct tissue niches rather than uniformly throughout the synovium. Early spatial analyses have mapped the localization of fibroblast, macrophage, endothelial, and lymphoid-rich regions in human RA synovium, supporting the concept that disease-associated cell states are shaped by local tissue context [11, 27]. However, many widely used spatial platforms, including 10x Visium, measure transcriptomes at the spot level rather than at true single-cell resolution [86, 92]. Therefore, current findings are best interpreted as tissue-organization maps, and specific cell-cell interaction claims require validation using higher-resolution spatial methods, imaging-based approaches, or orthogonal functional assays.

### Systemic Lupus Erythematosus and Lupus Nephritis

Systemic lupus erythematosus (SLE) is marked by broad heterogeneity, autoantibody production, immune-complex deposition, and prominent type I interferon-associated transcriptional programs [28, 93, 94]. Lupus nephritis (LN) has become a major focus of single-cell analysis because conventional biopsy interpretation cannot fully resolve interactions between infiltrating immune cells, resident renal cells, and clinical outcome [8, 29, 64, 95].

#### Cellular Heterogeneity and Biomarker Limitations in LN

Foundational single-cell surveys have shifted the conceptual framework of LN from an exclusive focus on leukocyte infiltration toward recognition of profound transcriptional reprogramming of resident renal cells [29]. Der et al. complemented this work by characterizing tubular epithelial and other resident kidney cells, demonstrating that interferon-stimulated programs are prominent within the tubular epithelium itself, rather than being restricted to infiltrating immune cells [64]. Together, these studies mapped complex local immune gradients and highlighted prominent interferon-stimulated gene (ISG) expressions within the tubular epithelium. However, their insights remain constrained by relatively small cohort sizes (*n* = 73 and 24, respectively) and limited longitudinal sampling, restricting the ability to establish the prevalence and stability of fine-grained cellular states across disease stages and treatment contexts. In addition, the populations represented in these cohorts may not capture the full spectrum of human genetic variation, including differences in allele frequencies across populations and geographic gradients [96], limiting assessment of the generalizability of these molecular signatures. These limitations also extend to non-invasive biomarker development: although urine-derived immune cells show qualitative transcriptomic concordance with tissue-infiltrating populations [29, 97], their diagnostic sensitivity, specificity, and prognostic reproducibility across independent cohorts and centers remain insufficiently established, precluding immediate clinical readiness.

#### Systemic B-cell Dysregulation and Distributed Tissue Programs

Integrated scRNA-seq and scBCR-seq have further illuminated B-cell dysregulation in SLE, identifying clonally expanded and atypical phenotypes [68, 73, 94]. Progress is currently hindered by redundant nomenclature - where terms like age-associated B cells (ABCs), DN2, and extrafollicular B cells describe overlapping cellular states that require analytical harmonization. Moreover, whether peripheral blood signatures capture critical pathogenic events remains an open question, given that locally expanded extrafollicular populations within target tissues may not enter circulation. This compartmentalization is superimposed on a pervasive, tissue-wide type I interferon program shared across immune and resident epithelial lineages rather than restricted to professional IFN-producing cells [29, 31, 64]. Although IFN-high and IFN-low patient endotypes are highly reproducible, their temporal dynamics and utility in guiding targeted biological therapies require further investigation.

Interpretation of these cellular and transcriptional states is further complicated by substantial variation in disease activity, immunosuppressive treatment, age, sex, ancestry, and intercurrent infection, all of which can influence circulating and tissue immune programs. In LN, differences in biopsy timing and renal compartment may further contribute to heterogeneity [32]. Consequently, apparent molecular endotypes should be distinguished from signatures reflecting treatment exposure, acute inflammatory activity, or clinical context [32, 33, 97].

### Type 1 Diabetes

Compared with RA and SLE, the single-cell evidence base in type 1 diabetes (T1D) remains less mature and relies heavily on cross-sectional human donor tissues and experimental animal models. Human pancreatic tissue is rarely accessible during disease evolution in living patients, and most human pancreatic studies rely on organ-donor programs, particularly the Network for Pancreatic Organ Donors with Diabetes (nPOD), while mechanistic evidence continues to derive substantially from murine models, especially the non-obese diabetic (NOD) mouse [9, 72]. These evidence sources provide complementary but non-equivalent information. Human datasets are predominantly cross-sectional, while NOD models permit longitudinal and experimental interrogation of mechanisms that cannot readily be tested in humans. Interpretation of donor-derived pancreatic signatures is also influenced by donor age, sex, disease duration, autoantibody status, cause of death, ischemia time, tissue preservation, and isolation procedures, which can introduce biological and pre-analytical variation independent of T1D pathobiology [34–36]. Accordingly, these evidence levels should not be interpreted interchangeably, particularly when inferring pathogenic trajectories or causal relationships. Direct longitudinal and functional evidence linking specific cellular states to disease progression in humans remains limited.

A major insight from single-cell and single-nucleus studies is that pancreatic β-cells in T1D exhibit altered stress, inflammatory, and regenerative states that may influence their vulnerability to immune-mediated destruction [9, 34, 72]. Fasolino et al. integrated transcriptomic and chromatin-accessibility data from nPOD donor islets and identified altered β-cell states together with ductal populations exhibiting dendritic-cell-like transcriptional features [56]. These observations support a model in which β-cell dysfunction is not simply a passive consequence of immune attack but may contribute to the cellular environment that sustains disease. However, the interpretation of β-cell stress signatures requires particular caution. Pancreatic islets are highly susceptible to dissociation-related perturbation, with enzymatic processing inducing immediate-early genes such as *FOS*, *JUN*, *JUNB*, and *HSPA1A* and unfolded-protein-response programs that can resemble genuine cellular stress [35, 36]. Single-nucleus RNA sequencing of frozen pancreas can reduce some dissociation-associated effects, although it provides less representation of cytoplasmic transcripts. In addition, donor-related factors, including cause of death and tissue-isolation conditions, can produce substantial baseline stress responses and potentially obscure or distort cytokine-responsive programs. Thus, β-cell stress is a biologically compelling feature of the T1D single-cell landscape, but its magnitude, temporal relationship to immune attack, and contribution to disease progression require further validation using orthogonal and longitudinal approaches [35, 36].

Human multimodal immune profiling of pancreas-draining lymph nodes has provided evidence consistent with, but not sufficient to establish, a hierarchical model of autoreactive CD8 + T-cell responses. In a cross-sectional HPAP organ-donor study using CITE-seq and high-parameter flow cytometry, Golden et al. identified an increased stem-like signature among memory CD8 + T cells in pancreas-draining lymph nodes from autoantibody-positive and T1D donors, including expansion of a CXCR3 + TOX− population with features including increased BACH2 and IL7R expression [37]. These observations are compatible with the NOD mouse model in which stem-like autoreactive CD8 + T cells can sustain downstream effector populations [38]; however, the human study did not establish antigen specificity, lineage relationships, islet trafficking, or diabetogenic function. Thus, the hypothesis that durable disease modification may require targeting precursor-like rather than terminal effector populations remains plausible but unproven in humans. The strongest functional evidence for this hierarchy remains murine, whereas the available human evidence is cross-sectional and based on a limited organ-donor cohort [38, 39].

Single-cell studies have also nominated peripheral biomarkers, including Th1-associated transcriptional programs in PBMCs, as candidates for progression risk and early disease activity [72]. Their clinical relevance remains uncertain because it has not been established whether such signatures provide predictive value beyond established auto-antibody-based and HLA-genotype-based risk stratification in adequately powered prospective cohorts. These findings should therefore be considered exploratory rather than clinically actionable.

Integration of scTCR-seq, scBCR-seq, and scATAC-seq has begun to provide additional resolution of autoreactive lymphocyte states and their regulatory programs. Convergent receptor features, recurrent sequence motifs, and chromatin-accessibility profiles have been used to nominate candidate mechanisms linking genetic risk to immune and β-cell states [40–42]. However, these studies generally involve relatively small cohorts and remain limited by tissue accessibility, disease-stage heterogeneity, and the difficulty of establishing functional causality in humans. Overall, single-cell studies have substantially expanded the cellular and molecular framework of T1D, but the field remains more hypothesis-generating than functionally validated compared with the stronger translational evidence available in RA and, for selected mechanisms, SLE.

### Multiple Sclerosis

MS is a chronic CNS inflammatory disease in which demyelination, neurodegeneration, and compartmentalized immune responses coexist [98, 99]. Single-cell and single-nucleus approaches permit interrogation of both infiltrating immune populations and CNS-resident cells across lesions, cerebrospinal fluid, blood, and experimental models [43]. As in T1D, access to active human lesion tissue is limited, and the evidentiary basis therefore differs substantially by sample source. Living-patient studies of blood and CSF provide clinically relevant information but only indirect access to CNS pathology, whereas donor or autopsy tissue provides direct cellular and molecular information from affected CNS compartments but is predominantly cross-sectional [44, 100]. Experimental autoimmune encephalomyelitis (EAE), in contrast, provides an experimentally tractable models for testing candidate mechanisms and cellular interactions relevant to CNS autoimmunity, but its findings cannot be assumed to represent human MS directly [101–103].

MRI-informed snRNA-seq has been a major methodological advance. Absinta et al. profiled demyelinated white matter lesion edges and identified “microglia inflamed in MS” (MIMS) and “astrocytes inflamed in MS” with neurodegenerative transcriptional programs concentrated at chronic active lesion rims [45]. Subsequent work combining snRNA-seq with MERFISH refined this by identifying CD8 + T cell niches at lesion rims associated with interferon-responsive and lipid-metabolism programs in microglia, with complementary in functional experiments in mouse EAE showing that perturbation of microglial lipid-handling can influence CNS inflammation [45, 46, 104]. Thus, human studies identify disease-associated cellular niches, whereas the EAE experiments provide mechanistic support for a candidate pathway. Importantly, the latter should be interpreted as functional evidence from an experimental model rather than as direct validation of the corresponding mechanism in human MS. Schirmer et al. independently reported selective vulnerability of upper-cortical CUX2 + projection neurons beneath meningeal inflammation [47]. Convergence of these studies on rim-associated pathogenic niches strengthens the framework, though broader replication across independent autopsy cohorts is still emerging. Whether lipid-laden “foam-cell-like” microglial states are causally pathogenic or simply reactive to myelin debris cannot be resolved from descriptive scRNA-seq alone [45, 104].

Single-cell studies of CSF in relapsing-remitting MS reveal expanded B cells and plasma cells, increased cell-type diversity, and activated T-cell trajectories. Schafflick et al. identified cluster-independent expansion of T follicular helper-like cells in CSF, supporting local T/B cell interaction within the CNS compartment [43, 48, 74]. Ramesh et al. identified clonally expanded B cells with antigen-experienced transcriptional phenotypes [49]. More recent comparative work shows that CSF B- and T-cell clonal expansion is observed across multiple neuroinflammatory diseases but is most prominent in MS [50]. Transcriptomic analyses have implicated CXCL12-CXCR4 signaling as a potential mechanism contributing to lymphocyte retention within the CNS compartment [43].

A crucial caveat is that CSF immune cells may not faithfully represent parenchymal disease: CSF predominantly samples meningeal and perivascular compartments, and CSF cell transcriptional profiles differ measurably from brain-resident populations [45, 47, 48]. CSF profiling complements but does not substitute for tissue-level analysis, particularly in progressive MS where compartmentalized inflammation behind an intact blood-brain barrier drives ongoing damage. A further challenge is the boundary between human and EAE data: most EAE models are predominantly CD4-driven whereas MS pathology features prominent CD8 involvement, EAE is typically monophasic or relapsing whereas a substantial fraction of MS patients progresses chronically, and lesion location and treatment responses diverge. Moreover, key features of human B-cell biology are only partially recapitulated in most EAE models. EAE cell states should accordingly be treated as candidate hypotheses for human disease rather than direct equivalents [45, 104].

## Shared Mechanisms Across Autoimmune Diseases

Although autoimmune diseases are clinically and anatomically heterogeneous, single-cell studies increasingly suggest that some pathogenic processes converge at the level of cellular states, signaling programs, and intercellular communication networks. Importantly, however, recurrence of a molecular signature across individual studies does not by itself establish a shared mechanism. Cross-disease convergence is more convincingly supported when the same cellular state or pathway is identified through direct integration or systematic comparison of independent diseases, tissues, or cohorts and is subsequently supported by functional or experimental evidence. Recent cross-disease single-cell studies provide such evidence, while also demonstrating that conserved programs coexist with disease-, tissue-, and cell-type-specific features. For example, Zhu et al. integrated more than 1.3 million immune cells from 239 samples across six autoimmune diseases and identified gene programs shared across diseases while retaining substantial disease-specific signatures. Similarly, Korsunsky et al. constructed a cross-tissue stromal atlas spanning rheumatoid arthritis, Sjögren’s syndrome, interstitial lung disease, and ulcerative colitis and identified shared pathological fibroblast phenotypes associated with distinct inflammatory tissue niches [51, 52].

These observations support a model in which autoimmune diseases are neither entirely mechanistically distinct nor driven by a single universal inflammatory program. Instead, they may be viewed as different manifestations of partially overlapping cellular and molecular networks, in which conserved inflammatory modules interact with disease-specific genetic, tissue, and environmental contexts. Accordingly, the following sections focus on evidence for reproducible cross-disease programs, while distinguishing genuinely shared mechanisms from signatures that are recurrent but context-dependent.

### Levels of Cross-disease Convergence

Recent single-cell and single-nucleus studies have begun to move beyond disease-specific characterization by directly comparing cellular states and molecular programs across autoimmune diseases [31, 79]. However, cross-disease “convergence” should not be treated as a single biological phenomenon. Similarity can arise at several levels, which differ substantially in evidentiary strength and mechanistic implication. To provide a rigorous evaluative framework, we distinguish four hierarchical levels of cross-disease convergence: (1) broad pathway-level overlap; (2) cell-type-specific pathway activity; (3) cross-disease cell-state similarity; and (4) shared regulatory networks and causal mechanisms (Table 2).

### Pathway-level Convergence and Cellular Context

Pathway-level overlap represents the broadest and most established form of convergence. Recurrent activation of type I interferon, NF-κB, and JAK-STAT signaling pathways has been recognized for decades through genetic, bulk-transcriptomic, and immunological studies predating single-cell technologies [105–107]. Accordingly, scRNA-seq should not be interpreted as having newly established these pathways as shared features of autoimmune disease. Rather, its principal contribution is to resolve the cellular and tissue compartments in which previously recognized pathways are activated. For example, while type I IFN response signatures are detectable across blood and affected tissues in SLE, RA, and other autoimmune diseases such as Sjögren’s syndrome, the biological consequences of such activation depend heavily on the cell type, microenvironmental niche, disease duration, and ongoing therapeutic exposure [31, 84]. Consequently, broad pathway convergence reflects common inflammatory milieus rather than identical disease-initiating mechanisms.

### Cell-type-specific Pathway Convergence

A second level is cell-type-specific pathway activity, in which single-cell analysis identifies the particular cellular populations expressing or activating a shared molecular program [52]. This provides substantially higher biological resolution than bulk-profiling, particularly when similar transcriptional programs are observed in homologous immune or stromal compartments across diseases [108]. For example, IFN-associated transcriptional programs may occur in myeloid populations in RA synovium and lupus nephritis kidney tissue, while also being detected in circulating immune populations in SLE [7, 22, 29, 31, 64]. Although these observational datasets confirm that homologous cell lineages engage comparable inflammatory cascades across diverse organ sites, they represent observational correlations; they do not establish that the host cells are biologically equivalent, nor that they share identical upstream triggers or disease-driving capacities.

### Cross-disease Convergence of Cellular States

A stronger claim is cross-disease cell-state similarity, referring to cellular populations that share not only a transcriptional program but also sufficiently comparable cellular identity, differentiation state, tissue context, and biological properties [109, 110]. This distinction is important because similar gene-expression modules can emerge through convergent activation of otherwise distinct cell populations. Whether transcriptionally aligned cell populations such as *ISG15 + CXCL10+* myeloid states or *CXCL13 +* peripheral helper T cells observed across synovial, renal, and central nervous system lesions - represent genuinely homologous, disease-associated cellular lineages or parallel reactive states driven by localized inflammatory cues remains an open question. Cross-sectional observational data cannot resolve this distinction, as differences in tissue dissociation protocols, disease stage, previous therapeutic exposure, and technical batch effects frequently confound direct cross-disease integration [109, 110].

### Regulatory-network Convergence and Mechanistic Uncertainty

The highest level considered here is shared regulatory-network or mechanistic convergence, in which similar transcription-factor activity, chromatin accessibility, enhancer-gene relationships, or gene-regulatory circuits across diseases. Single-cell multi-omics approaches can nominate such relationships by integrating transcriptional and epigenomic information [111–113]. However, computationally inferred regulatory similarity does not establish that the same circuit is causally required in each disease. Demonstrating genuinely shared regulatory mechanisms requires orthogonal experimental evidence, ideally including perturbation studies that test whether disruption of the candidate regulator or circuit produces comparable phenotypic effects across disease contexts.

### A Hierarchy of Cross-disease Molecular Convergence

Overall, cross-disease convergence is strongest and best established at the pathway level, whereas cell-type-specific activity provides necessary resolution to map cell-type-specific activity and nominate conserved cell states. Importantly, shared molecular signatures are strongly shaped by cellular and tissue context, so molecular convergence alone should not be interpreted as evidence of shared pathogenic mechanisms or equivalent therapeutic responses. Future cross-disease single-cell studies should therefore specify the level of convergence being claimed and distinguish pathway similarity, cell-type-specific pathway activity, cell-state similarity, regulatory-network similarity, and functional equivalence [31, 79, 114]. Table 2 summarizes the proposed hierarchy of cross-disease convergence, highlighting the increasing uncertainty from pathway-level to cell-state- and regulatory-network-level similarity and the implications for therapeutic interpretation.

**Table 2:** Levels of cross-disease convergence identified by single-cell studies in autoimmune diseases

Level of convergence	Key concept / definition	Representative examples across RA, SLE/LN, T1D and MS	Evidence and key uncertainty	Implication for therapeutic translation	Representative references
Pathway-level	Shared inflammatory signaling pathways are activated across diseases	Type I IFN, JAK-STAT, NF-κB and related inflammatory signaling programs recur across autoimmune diseases	**Strongest and most established level of convergence**. However, shared pathway activation does not establish shared cell identity, pathogenic function, or therapeutic response	Supports investigation of shared pathway-targeted therapies, but pathway overlap alone does not predict cross-disease efficacy	[31, 84, 105, 106, 107]
Cell-state-level	Similar immune or stromal cell states exhibit overlapping transcriptional programs across diseases and tissues	IFN-responsive myeloid states in SLE/LN, inflammatory myeloid states in RA synovium, and myeloid activation programs in MS lesions	**Emerging evidence**. Similar transcriptional states may reflect conserved cell states or distinct cells responding to similar inflammatory cues	May enable cell-state-directed therapies if conserved states are independently and functionally validated	[31, 52, 109]
Regulatory-network-level	Transcription-factor activity and gene-regulatory programs are potentially conserved across diseases	Candidate shared regulatory programs involving STAT1, IRF5, IRF8 and related inflammatory transcriptional regulators	**Limited and largely inferential**. Similar TF activity does not establish conserved regulatory architecture or causal function.	Could identify precision regulatory targets, but functional validation is required before translation	[111, 112, 113]
Tissue-context dependence	Shared molecular programs are interpreted within disease-specific cellular and tissue environments	IFN programs in circulating immune cells versus renal, synovial, pancreatic or CNS-resident compartments	**Strong conceptual support**,** but important context dependence**. The same pathway can generate different outputs depending on cell identity, tissue, and disease stage	Therapeutic transferability depends on **where and when** a target is expressed, not simply whether the pathway is activated	[64, 79, 109]
Homologous state vs. parallel activation	Cross-disease similarity may reflect genuinely conserved cell states or independent activation of distinct cells	Similar IFN-responsive myeloid signatures across RA, SLE/LN and MS may not represent equivalen[t biological states	**Major unresolved issue**. Transcriptomic similarity alone cannot establish cellular homology or shared pathogenic function	Requires harmonized atlases, consistent cell ontologies, spatial information, and functional validation before therapeutic equivalence can be inferred	[31, 52, 79, 109]

## Translational Relevance

Single-cell profiling can in principle support biomarker discovery for diagnosis, prognosis, and treatment selection [6, 79, 115]. However, a clear distinction must be drawn between exploratory cellular phenotyping and establishing clinically validated biomarkers. Identifying an enriched pathogenic cell state or generating a disease-associated gene signature represents only the initial hypothesis-generating phase of biomarker discovery. Moving from these exploratory signatures to routine clinical practice requires demonstrating analytical validity (reproducibility, sensitivity, and standardization), clinical validity (robust correlation with defined clinical endpoints in independent, multi-center cohorts), and clinical utility (actionable impact on patient outcomes beyond standard-of-care metrics). A realistic assessment therefore requires distinguishing conceptual advances from biomarkers that have undergone prospective clinical validation.

### Treatment Response Prediction in Rheumatoid Arthritis

One of the most clinically advanced application of synovial molecular profiling in RA is the R4RA randomized trial, which compared rituximab and tocilizumab in 164 patients with RA who had inadequate response or intolerance to conventional synthetic DMARDs and at least one TNF inhibitor [116]. Thus, participants were TNF-inhibitor inadequate responders rather than patients with established multidrug-refractory disease at study entry. The trial stratified treatment according to baseline synovial B-cell status and prospectively evaluated whether tissue molecular features could inform differential therapeutic responses [116, 117]. These findings provided important proof of principle that synovial molecular features may enrich for differential therapeutic responses, although they should not be interpreted as definitive evidence of treatment selection efficacy.

Subsequent molecular analysis of the R4RA biopsies provided further insight into the biological basis of treatment response. Patients with low or absent synovial B-cell molecular signatures showed lower response to rituximab compared with tocilizumab, and machine-learning models trained on synovial transcriptomic features achieved AUC values of 0.74 for rituximab response, 0.68 for tocilizumab response, and 0.69 for multidrug resistance [116]. A fibroid pauci-immune pathotype emerged as a refractory endotype associated with non-response to multiple biologic classes including TNF inhibitors, suggesting that currently available biologic therapies may provide limited benefit for this subgroup.

The R4RA trial provides important prospective randomized evidence that tissue-based synovial molecular features can enrich for differential therapeutic responses in rheumatoid arthritis [116, 117]. However, its reported AUC values indicate predictive enrichment rather than deterministic classification, meaning that these signatures cannot yet replace standard clinical algorithms. Importantly, R4RA relied on bulk synovial transcriptomics and histopathology rather than scRNA-seq; therefore, it should be interpreted as prospective randomized evidence supporting tissue-based molecular stratification rather than prospective validation of single-cell derived biomarkers [116]. Whether cell-state-resolved classifications derived from scRNA-seq provide superior or independent predictive power over bulk transcriptomic or histopathological frameworks remains unproven. Demonstrating that high-dimensional single-cell states offer true incremental clinical utility will require dedicated prospective evaluation within randomized controlled trials [21]. Furthermore, routine translation of tissue-based molecular biomarkers faces significant translational barriers, including the constraints of image-guided biopsies, assay standardization, multi-center reproducibility, turnaround times, and health-economic justification [6].

### Translational Opportunities in LN, MS, and T1D

In LN, single-cell-informed urine biomarkers may offer non-invasive longitudinal monitoring and reduce reliance on repeated kidney biopsy [29, 97]. As discussed in Sect. 4.2, current evidence supports the conceptual feasibility of this approach but does not yet establish clinical performance characteristics. Urine single-cell profiling should therefore be regarded as a research platform with considerable translational potential rather than a clinically validated assay. Prospective studies evaluating diagnostic performance, longitudinal stability, and inter-center reproducibility will be required before routine clinical implementation can be considered [29, 30, 97].

In MS, CSF single-cell profiling may complement imaging by providing a dynamic molecular view of compartmentalized inflammation [48–50]. The primary translation question is whether CSF cellular signatures can refine prognosis, predict treatment response (especially therapies targeting B cells), or identify patients transitioning to progressive disease earlier than current methods. While initial descriptive findings are promising, available cohorts remain largely cross-sectional, retrospective, and underpowered. Logistical constraints - including invasive lumbar punctures, low CSF cell yields, and the lack of automated, clinical-grade microfluidic workflows - severely restrict direct clinical implementation. Moreover, repeated lumbar puncture and standardized processing pipelines pose practical challenges that may limit the routine use of CSF single-cell profiling in clinical practice [48–50].

In T1D, peripheral immune signatures and receptor-based analyses may improve risk stratification and guide antigen-specific or tolerance-inducing interventions. The clinical context is challenging because T1D is rare, requires long prediction horizons, and is already supported by well-validated autoantibody-based and HLA-genotype-based risk stratification. Therefore, any single-cell-derived biomarker will need to demonstrate incremental predictive value beyond these existing tools. In addition, most proposed cellular signatures remain at the discovery or proof-of-concept stage and have not been established as clinically validated biomarkers. Thus, translation will require independent replication, standardized and analytically validated assays, evaluation in clinically representative and longitudinal cohorts, and demonstration of clinical validity and utility [118–120].

### Drug Mechanism of Action and Target Discovery

Beyond patient stratification, single-cell methods are increasingly useful for studying drug mechanism of action and resistance. Longitudinal tissue or blood profiling can reveal whether a given therapy eliminates pathogenic populations, partially suppresses them, or shifts them into alternate disease-supporting states [7, 115]. The Alivernini et al. study of remission-associated synovial macrophages [7], described in Sect. 3.1, exemplifies this approach: effective therapy may need to support pro-resolving programs in addition to suppressing inflammatory cells. Single-cell perturbation methods – such as Perturb-seq and related CRISPR-coupled scRNA-seq approaches-are emerging as a route to systematically dissect immune regulatory circuits and validate candidate drug targets [121–123]. By applying Perturb-seq to human immune cells, investigators have begun linking functional regulators of inflammatory pathways to non-coding genetic variants identified in genome-wide association studies (GWAS) [122]. While not yet routinely applied to primary patient samples in autoimmune disease, these methods provide a critical link between descriptive single-cell association studies and the functional validation required to translate observations into therapeutic targets [122, 124].

## Current Challenges

**Fig. 3:**
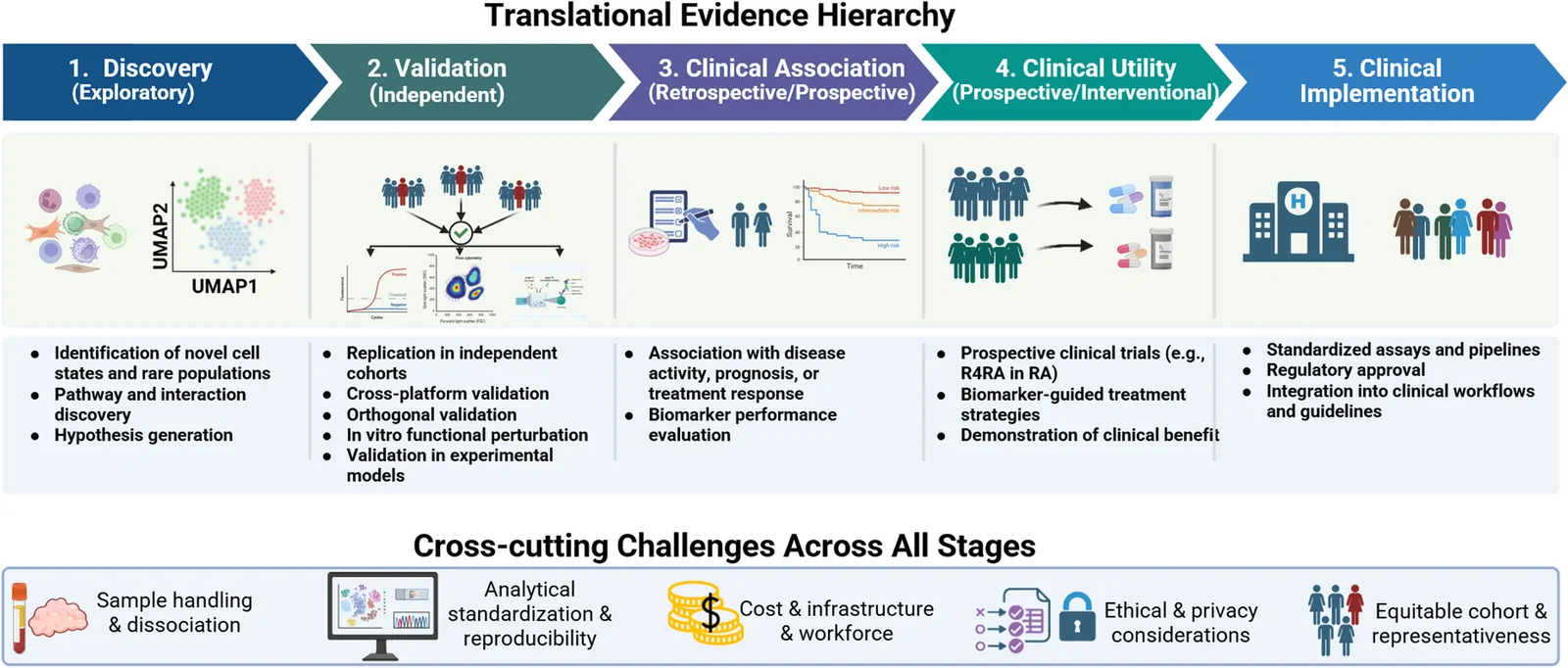
Translational evidence framework and current challenges for clinical implementation of single-cell transcriptomics in autoimmune diseases (Created by BioRender)

A major challenge for translating single-cell discoveries into clinical practice is the gap between biological insight and demonstrated clinical utility. Single-cell and spatial studies have identified numerous candidate pathogenic cell states, molecular pathways, cellular interactions, and biomarkers in autoimmune disease, yet relatively few findings have progressed beyond discovery and initial clinical association. The top part of Fig. 3 presents a translational-evidence hierarchy spanning five levels: discovery, independent validation, clinical association, clinical utility, and clinical implementation. These levels represent progressively stronger evidence, from identifying candidate biological features and establishing their reproducibility to demonstrating their association with clinically meaningful outcomes, prospective value for clinical decision-making, and ultimately reliable use in routine practice. The hierarchy therefore highlights a central challenge for the field: biological plausibility and reproducibility alone are insufficient to establish clinical usefulness, and substantial evidence gaps remain between single-cell discovery and implementation in patient care.

The figure comprises two parts. Top: Translational evidence hierarchy. The hierarchy illustrates the progression from discovery and exploratory studies through independent validation, clinical association, clinical utility, and clinical implementation. Bottom: Challenges to translation. The major challenges encountered across the translational pathway include sample collection and processing, analytical standardization and reproducibility, cost and infrastructure requirements, workforce and expertise, ethical and privacy considerations, and the recruitment of representative and equitable patient cohorts. Together, the two panels highlight both the stages required to advance cellular and molecular biomarkers toward clinical practice and the barriers that may hinder successful translation.

Several interconnected technical, biological, analytical, clinical, and operational barriers contribute to this translational gap. As summarized in the lower part of Fig. 3, these challenges span the entire research workflow, from sample acquisition and processing to analytical standardization and reproducibility, as well as the cost and infrastructure required for large-scale studies, ethical and privacy considerations, and the representativeness and equity of study populations. These factors can influence not only the quality and interpretability of single-cell datasets but also the reproducibility, generalizability, and feasibility of translating discoveries across cohorts and clinical settings. The following subsections consider these challenges individually and discuss their implications for the application of single-cell RNA sequencing in autoimmune disease research.

### Sample Handling and Dissociation

A primary hurdle in translating single-cell transcriptomics to clinical utility lies in pre-analytical sample preparation and enzymatic dissociation. Tissue dissociation can selectively lose fragile cell types, reduce viability, and induce stress-response programs that complicate biological interpretation. Immediate-early genes (*FOS*,* JUN*,* JUNB*), heat-shock genes (*HSPA1A*), and unfolded protein responses can be induced by enzymatic dissociation and mimic genuine stress signatures [63, 125, 126]. The problem is not unique to pancreatic islets (Sect. 4.3): similar artifacts confound disease-state comparisons in other solid tissues if dissociation protocols differ between cases and controls. snRNA-seq of frozen tissue mitigates these dissociation-induced artifacts and is increasingly used for tissues that are difficult or susceptible to conventional dissociation, providing reduced representation of cytoplasmic transcripts and lower sensitivity for immune receptor sequences [36, 127]. Standardized dissociation protocols comparable to those developed by AMP for synovial tissue are essential for cross-study comparability, and journals could reasonably require explicit reporting of dissociation conditions, viability metrics, and stress-gene signatures [21, 128].

### Computational Analysis and Reproducibility

Beyond sample handling, the analytical pipeline introduces considerable technical variability. Single-cell datasets are sparse, noisy, and highly sensitive to dropout, batch effects, and parameter choices [54, 129–132]. Multiple integration methods - including Harmony, Seurat, scVI, Scanorama, LIGER - are available, but no framework is universally optimal; performance depends on datasets structure, biological objective, and integration design [129, 132]. Interpretation is further complicated by clinical and pre-analytical covariates, including treatment exposure, disease activity, age, sex, ancestry, infection status, ischemia time, cryopreservation, processing center, and sequencing batch, which can contribute to transcriptional variation independently of disease biology [133]. Methods should therefore be evaluated not only by batch mixing but also by preservation of cell identity, disease-associated variation, and biologically meaningful trajectories. These computational choices can directly influence downstream biological interpretation, including cell-state identification and cross-cohort comparisons, because differences in cohort composition, technical platforms, and reference representations may affect estimates of disease-associated cell abundance and marker specificity [129, 132]. This is particularly important in autoimmune studies, where disease status, tissue, treatment, and donor effects may be strongly structured. Overcorrection can erase genuine disease-associated states, whereas under-correction can leave technical artifacts that are misinterpreted as biology. Reference-query approaches may facilitate standardized annotation and mapping, but reliance on healthy reference atlases can also obscure disease-specific states that are absent from the reference.

Cell-type annotation introduces additional reproducibility challenges. Manual marker-gene annotation is the historical standard but remains subjective and difficult to harmonize. Automated reference-based tools (Azimuth, CellTypist, scArches) increasingly support standardized cell-type calls [134, 135], but as discussed in Sect. 3.4, autoimmune tissues contain disease-associated states absent from healthy reference atlases. Automated assignments therefore require biological review and, where necessary, re-annotation of disease-specific states. Community efforts to develop harmonized, disease-specific reference atlases and standardized evaluation frameworks will be essential for improving reproducibility without obscuring clinically relevant cellular heterogeneity. Clinical and pre-analytical variables can further interact with computational batch effects. Differences in processing center, tissue handling, cryopreservation, library preparation, sequencing batch, and data-processing pipelines may introduce variation that is difficult to distinguish from biological heterogeneity, emphasizing the need for comprehensive metadata and appropriate batch-aware study designs.

### Reproducibility, Cost, and Clinical Implementation

Routine clinical adoption requires robust cross-center reproducibility, cost-efficiency, and standardized quality-assurance frameworks. At present, reproducibility across centers and platforms remains challenging because tissue handling, library preparation, sequencing, and computation analysis can each introduce sources of variation. Moreover, many proposed single-cell biomarkers and patient stratification signatures remains supported primarily by relatively small, deeply phenotyped discovery cohorts rather than large prospective populations, and independent replication across cohorts and platforms is the exception [136]. Per-sample costs - depending on sequencing depth, sample complexity, and platform - are substantially higher than bulk RNA-seq or standard immunohistochemistry, limiting feasibility for large clinical cohorts [5, 54, 115, 131]. Regulatory frameworks and quality-assurance standards for clinical implementation of single-cell diagnostics are still evolving and remain less mature than those established for conventional molecular assays. Moreover, clinical laboratory accreditation would require standardized analytical pipelines, performance specifications, and external quality assessment programs that do not yet exist [5, 6].

### Ethical and Privacy Considerations

Finally, single-cell and multimodal sequencing datasets introduce heightened regulatory and privacy challenges. Ethical considerations deserve greater attention than they typically receive. In particular, sequencing data should not be considered inherently anonymous. Genomic variants can be inferred from scRNA-seq reads with sufficient accuracy in some datasets to facilitate subject re-identification when combined with external genomic resources [137, 138]. Privacy risks may be further increased when scRNA-seq is combined with scTCR-seq or scBCR-seq, because immune receptor repertoires are highly individualized and can provide additional identifying information when integrated with other datasets [137]. Even relatively small numbers of informative variants may facilitate re-identification under certain circumstances [138, 139]. These issues have important implications for informed consent, public-repository data sharing, and future diagnostic applications. Consequently, the open-data model that has accelerated single-cell biology may need to be balanced with controlled-access repositories and emerging federated analysis frameworks that enable collaborative analysis without unrestricted transfer of raw data.

Beyond privacy, equitable cohort representation is essential. Many current autoimmune single-cell atlases have been generated predominantly from North American and European cohorts [21, 29, 45], reflecting broader patterns of underrepresentation in genomic research [140, 141]. However, differences in molecular signatures and treatment-prediction models cannot be attributed to genetic variation alone. Environmental and social determinants including diet, smoking, infections, medication exposures, socioeconomic conditions, and healthcare access can shape immune and tissue-specific gene-expression programs and contribute to variability across genetically similar populations [142]. These interacting influences, partly captured by the exposome, may limit reproducibility across cohorts and healthcare settings [143]. Future studies should therefore evaluate biomarker performance and treatment-response models in the context of genetic ancestry, environmental exposures, sex, demographic and social factors, disease subtype, and access to care. Precise use of terms such as sex, race, and genetic ancestry is also essential, as they represent distinct biological, social, and population-genetic dimensions and should not be used interchangeably [140, 141]. This rigor is essential for robust interpretation, public trust, and equitable precision medicine.

Overcoming these inequities requires moving beyond post hoc validation toward prospective, equity-by-design approaches informed by established genomic initiatives. The Human Cell Atlas Equity and Diversity Working Group, H3Africa, and the NIH *All of Us* Research Program illustrate complementary models of community-engaged recruitment, regional research capacity building, inclusive reference development, and responsible data stewardship [144–148]. In rheumatology, multi-ethnic cohorts such as the California Lupus Epidemiology Study (CLUES) and the AMP RA/SLE program further demonstrate the value of broad population representation and harmonized clinical and molecular profiling [149, 150]. For single-cell autoimmune research, these principles support deliberate recruitment of underrepresented populations, cross-population validation of biomarkers and predictive models, harmonized clinical and biospecimen metadata, and equitable involvement of local investigators in study design, analysis, and data governance. Such approaches are essential to establish whether single-cell biomarkers generalize across populations and clinical settings rather than merely reproducing findings within the populations in which they were discovered. Ultimately, the successful clinical translation of single-cell technologies will depend not only on analytical performance but also on robust ethical governance. Safeguarding genomic privacy, ensuring equitable representation and cross-population validation, promoting transparency of computational and artificial intelligence methods, and establishing appropriate frameworks for data sharing and management of incidental findings will be essential to maintain public trust and to ensure that the benefits of single-cell medicine are distributed fairly across populations. Addressing these challenges proactively should be regarded as a prerequisite for responsible implementation rather than as a secondary consideration following technological development [140, 151, 152].

## Future Directions

The next phase of autoimmune single-cell research will likely be defined by four priorities: multimodal integration, longitudinal study design, functional perturbation, and clinical standardization. Progress will determine whether the field continues to generate descriptive atlases or transitions to mechanism-anchored, clinically actionable biology.

### Multimodal and Spatial Integration

Combining transcriptomic, proteomic (CITE-seq), epigenetic (scATAC-seq), T-cell and B-cell receptors (scTCR/scBCR-seq), and spatial data within the same study enables more complete definitions of pathogenic circuits than any single modality [15, 153, 154]. Multimodal integration is technically demanding, but consortium studies including AMP Phase 2 in RA and chronic active MS atlases demonstrate that linking transcriptional states to chromatin accessibility, surface phenotype, and tissue location provides mechanistic depth unattainable from any modality alone. Higher-resolution spatial technologies such as MERFISH, Slide-seqV2, Stereo-seq, Xenium, and CosMx are increasingly complementing spot-based platforms and promise improved resolution of cellular interactions [155, 156]. Future studies should be designed from the outset with multimodal integration in mind, rather than as transcriptome-only experiments with retrospective layering of additional data.

### Longitudinal Sampling and Dynamic Disease Models

Longitudinal sampling is essential for understanding how disease-associated cell states emerge, fluctuate, respond to treatment, and contribute to relapses or remission. Most single-cell studies remain cross-sectional, limiting direct inference about temporal dynamics and causality. Serial blood sampling is feasible and used in RA and SLE pilot treatment-response studies [29, 157], but serial tissue sampling-kidney, synovium, CNS-is logistically and ethically challenging. Strategies include opportunistic sampling at clinically indicated procedures, non-invasive proxies such as urine in LN [29] and CSF in MS [48, 49], and computational trajectory inference from cross-sectional snapshots-the last requiring careful validation against actual longitudinal data because pseudotime ordering can be misleading in chronic relapsing diseases [158–160].

### Functional Perturbation and Target Validation

Single-cell perturbation methods-Perturb-seq, CROP-seq, Perturb-ATAC-will bridge the gap between descriptive cell-state biology and causal mechanism [121, 122, 161, 162]. Applied to primary or stem-cell-derived immune cells, these methods allow systematic interrogation of candidate disease genes and drug targets with single-cell readouts. In autoimmune disease, where genome-wide association studies have nominated hundreds of risk loci whose mechanisms remain unclear [163], single-cell perturbation offers a tractable path to assign genetic risk to specific cell types and transcriptional programs [122, 123]. Recent compressed and scalable Perturb-seq variants enable genome-scale screens at reasonable cost [122, 123]. Integration with disease atlases and human genetics will be a defining methodological development for the next several years.

### Clinical Standardization and Mechanism-based Reclassification

Standardized workflows will be required before single-cell-derived biomarkers can enter routine diagnostics. These include pre-analytical standardization of sample collection, tissue dissociation, cell viability, and preservation; analytical standardization of quality control, integration, reference-based annotation, and reproducibility assessment; and clinical-grade reporting with defined performance metrics. Consortium-led reference atlases, including resources from the Human Cell Atlas and HuBMAP, together with annotation and mapping tools such as Azimuth and CellTypist, provide an important foundation for harmonized analysis [69, 79, 136, 164]. However, their integration into accredited clinical laboratory workflows and regulatory-grade diagnostic pipelines has not yet been demonstrated.

The paradigm of autoimmune disease classification is shifting from organ-centered phenotypes toward mechanistically defined molecular endotypes. If prospectively validated, cell-state-resolved stratification will enable rational treatment selection by matching targeted therapies directly to dominant pathogenic programs rather than clinical diagnoses. While the R4RA trial demonstrated that tissue-derived histopathological and bulk transcriptomic features can inform therapeutic stratification in rheumatoid arthritis [116, 117], expanding this concept to single-cell resolution remains a critical translational bottleneck. Realizing this mechanism-based framework demands umbrella or platform trial designs that allocate therapies by molecular endotype, alongside academic-industrial partnerships and collaborative regulatory frameworks that formalize endotype-driven stratification in clinical decision-making.

## Conclusion

scRNA-seq has substantively transformed autoimmune disease research by resolving cellular heterogeneity, tissue-specific organization, and dynamic cellular states that are largely inaccessible to bulk analyses. In RA, SLE/LN, T1D, and MS, single-cell and spatial studies have revealed pathogenic immune and stromal states, clarified local cellular interactions, and generated hypotheses about disease mechanisms, biomarkers and therapeutic targets [7, 8, 29, 45]. Cross-disease comparisons further suggest shared inflammatory programs-most consistently type I interferon signaling, chemokine-mediated recruitment, and aberrant tissue-immune crosstalk, although the strength of such convergence claims remains limited because truly harmonized cross-disease single-cell analyses are still relatively uncommon [7, 29, 31].

The major challenge now is to determine which cellular states are reproducible, temporally informative, mechanistically causal, and clinically actionable. Current evidence remains constrained by relatively small and geographically limited cohorts, treatment and disease-stage heterogeneity, technical and analytical variability, and limited prospective validation. Among these limitations, the predominantly cross-sectional design of many single-cell studies represents a major structural obstacle to causal inference [158, 160]. A single time point can identify cellular states associated with disease, severity, tissue damage, or treatment response, but cannot by itself establish whether these states precede disease, emerge as a consequence of inflammation or treatment, or persist as part of the disease process. Longitudinal sampling across disease initiation, progression, remission, relapse, and treatment is therefore critical for establishing temporal relationships and distinguishing persistent, disease-relevant programs from transient or context-dependent responses [157].

Realizing the translational potential of single-cell technologies requires moving beyond discovery-stage research, as a substantial gap remains between the current evidence base and clinical readiness. Addressing this gap will require longitudinal studies across diverse populations, improved reproducibility across cohorts and technologies, harmonized analytical frameworks, and integration of single-cell measurements with spatial, multimodal, clinical, and functional perturbation data [136]. Prospective validation will ultimately be necessary to establish whether single-cell-derived signatures can predict disease trajectories, identify treatment-responsive states, or guide therapeutic selection [116, 165].

Importantly, this gap in clinical validation does not diminish the established value of scRNA-seq for basic and mechanistic discovery. By revealing previously unrecognized cellular states, mapping tissue-specific interactions, and providing a framework for understanding how cellular programs evolve during disease, scRNA-seq has become a pivotal tool for elucidating autoimmune pathogenesis [7, 8, 29, 45]. The next phase of the field should therefore move beyond increasingly detailed cellular atlases toward longitudinal, mechanistically validated, and clinically informative models of disease, in which cellular states and trajectories complement conventional clinical phenotypes to advance precision medicine.

## Data Availability

No datasets were generated or analysed during the current study.

## Abbreviations

Abbreviation: Full term
CITE-seq: Cellular indexing of transcriptomes and epitopes by sequencing
AMP: Accelerating Medicines Partnership
AS: Ankylosing Spondylitis
CNS: Central Nervous System
CSF: Cerebrospinal Fluid
CTAPs: Cell-Type Abundance Phenotypes
EAE: Experimental Autoimmune Encephalomyelitis
FFPE: Formalin-Fixed Paraffin-Embedded
HCA: Human Cell Atlas
HPAP: Human Pancreas Analysis Program
HuBMAP: Human BioMolecular Authentication/Atlas Program
MHC: Major Histocompatibility Complex
LN: Lupus nephritis
MS: Multiple sclerosis
RA: Rheumatoid arthritis
NK: Natural Killer (cell)
NOD: Non-Obese Diabetic (mouse)
PBMC: Peripheral Blood Mononuclear Cell
TCR: T-Cell Receptor
BCR: B-Cell Receptor
Treg: Regulatory T (cell)
Tn: Naïve T (cell)
scATAC-seq: Single-cell assay for transposase-accessible chromatin using sequencing
scBCR-seq: Single-cell B-cell receptor sequencing
scRNA-seq: Single-cell RNA sequencing
scTCR-seq: Single-cell T-cell receptor sequencing
SLE: Systemic lupus erythematosus
snRNA-seq: Single-nucleus RNA sequencing
T1D: Type 1 diabetes
ST: Spatial transcriptomics
TNF: Tumor Necrosis Factor
IFN: Interferons
TF: Transcriptional Factor
